# Evaluating Risk and Benefit Sensitivity for Cognitive Treatments

**DOI:** 10.1007/s41465-025-00319-3

**Published:** 2025-03-22

**Authors:** Kianté A. Fernandez, Brian A. Erickson, Joseph W. Kable, Roy H. Hamilton, John D. Medaglia

**Affiliations:** 1 Department of Psychology, University of California, Los Angeles, CA USA; 2 Department of Psychological & Brain Sciences, Drexel University, Philadelphia, PA USA; 3 Department of Neurology, University of Pennsylvania, Philadelphia, PA USA; 4 Department of Psychology, University of Pennsylvania, Philadelphia, PA USA

**Keywords:** Decision making, Experimental neuroethics, Neuromodulation, Risk preferences

## Abstract

Ethical judgments require clinicians, researchers, research participants, and patients to weigh risks and benefits. Novel treatments for cognitive deficits are rapidly emerging, but little is known about how individual differences in risk and benefit sensitivity influence ethical judgments to administer treatments. The public plays important roles as citizens, taxpayers, and consumers of cognitive treatments, yet little is known about how they evaluate risks and benefits in ethical judgments. We examined the influence of risk and benefit sensitivity on the public’s choices about treating cognitive dysfunction. We administered surveys, cognitive measures, and an ethical judgment paradigm to 425 participants recruited via Amazon Mechanical Turk. Participants were asked to choose whether to recommend a hypothetical cognitive treatment with varying degrees of risks and benefits across seven different cognitive domains. We expected participants to be more risk-sensitive than benefit-sensitive, especially when evaluating treatments that influence cognitive functions central to personal identity such as mood, self-control, and long-term memory. Unexpectedly, participants were slightly more sensitive to benefits and showed inter-domain stability across cognitive dysfunctions. Our results suggest that risks and benefits influence whether the public might recommend cognitive treatments. The relatively higher weight placed on benefits could be explained by prominent theories of decision-making under risk. Overall, this study suggests that judgment tasks can be adapted to study psychological ethical choices about treatments for cognitive deficits. Further study of individual variation in risk and benefit sensitivity and their influence on real-world ethical choices about cognitive repair could inform frameworks to enhance optimal neuroethical decision-making.

## Introduction

Cognitive deficits can be treated by modifying the nervous system. Cognitive treatments use methods such as talk therapy, electric stimulation, chemical agents to advanced techniques such as deep brain implants, and closed-loop adaptive algorithms, which selectively target mechanisms to address cognitive and motor dysfunctions (Ezzyat et al., 2018; Johnson et al., 2016; Kim et al., 2017). As technological advancements expand our ability to modify cognitive functions, ethical considerations become increasingly crucial. Ongoing neuroethical debates inform our ability to prevent harm but also respect autonomy, agency, and personhood (Cabrera et al., 2014; Medaglia et al., 2017, 2020). However, as we continue to anticipate and address ethical questions raised by cognitive treatments, the psychological factors influencing individuals’ decisions to influence cognition and treat deficits are still largely unknown.

As these technologies and the relevant debates about modifying cognition emerge (Amadio et al., 2018), the public plays a crucial role as a key stakeholder. The public contributes to funding treatments for cognitive deficits research through taxation and some members of the public become participants and patients in research on treatments for cognitive deficits (Li et al., 2022). How individuals weigh the risks and benefits of treating cognition could determine whether or not they participate in effective treatments. A sizable portion of the public expresses apprehension about implanted neural devices, with 69% expressing that they are at least somewhat concerned (Funk et al., 2016; Klein et al., 2023). While studies have begun to reveal public attitudes toward treatments for cognitive deficits, we know little about what drives public worry.

Prior studies have provided some insight into what drives public attitudes toward interventions on cognitive function. When it comes to interventions aimed at enhancing cognition, among the public, people report being more reluctant to enhance aspects they consider fundamental to their identities (Riis et al., 2008). Additionally, research suggests that public opinions are influenced by the perceived effects and moral implications of using technologies to influence cognition. For instance, in a trinational survey, respondents reported both enthusiasm and worry about brain-computer interface technology depending on the domain of application and sociodemographic factors (Sample et al., 2019). Research on cognitive enhancement has also demonstrated that public attitudes are influenced by framing effects (Schelle et al., 2014). Furthermore, factors like peer pressure, the influence of authority figures (Conrad et al., 2019; Dinh et al., 2020), competition (Greely et al., 2008; Partridge et al., 2012), moral relevance (Cabrera et al., 2015a; Faber et al., 2015; Scheske and Schnall, 2012), familiarity with enhancement devices (Bell et al., 2013), expertise (Banjo et al., 2010; Fitz et al., 2014), and the domain of cognitive enhancement (Haslam et al., 2020) can all influence public attitudes. Moreover, the public is sensitive to the difference between cognitive enhancement and treatments. People are more willing to endorse repairing cognitive functions than enhancing them and are more hesitant when they consider the morality of a particular intervention first (Haslam et al., 2020; Medaglia et al., 2019a). Among patients, patients’ reduced compliance for cognitive treatments is caused by psychological factors (Marrero et al., 2020; Mohr et al., 2010; Velligan et al., 2017) and sociodemographic factors (Barrett et al., 2008). Overall, the evidence suggests that for cognitive enhancements and treatments, factors such as framing, evaluation, mechanism of action, and the specific cognitive target of interventions can significantly influence individuals’ willingness to use them.

Despite broader research themes, whether neuroethical principles loom large in public judgments is relatively less studied. A fundamental neuroethical guiding principle, *safety*, requires risks to be rigorously assessed and carefully weighed against the likely benefits of research and treatments. Psychological risk^1^ and benefit sensitivity have been studied in medical decision-making for many years, including in choices involving treatment recommendations (Hauber et al., 2013; Rasiel et al., 2005; Waters et al., 2007). Research suggests that individual variability in risk and benefit sensitivity is indeed evident in consumer and clinician decision-making (Galizzi et al., 2016; Nadler and Reiner, 2010; Zhang et al., 2019). If individual variability in risk and benefit sensitivity exists across consumer and clinician contexts, it is possible this variability might also exist in ethical judgments about treatments for cognitive deficits. If such variability is evident when the public or others evaluate treatments, individual differences could have real-world consequences. For instance, risk-tolerant individuals could take individual actions or suggest policies that are more likely to result in harm (Eichler et al., 2013). In addition, disagreements about treatments for cognitive deficits could motivate either enthusiasm or antagonism to ongoing research efforts, influencing funding, participation, and public understanding. However, although neuroethicists have called for more quantitative approaches to explore issues pertinent to neuroethics, quantitative approaches to studying risk and benefit sensitivity have not been applied to ethical judgments about cognitive treatment (Fernandez et al., 2022; Fitz et al., 2014; Reiner, 2019; Specker et al., 2017).

To address this gap, we examined whether risk & benefit sensitivity could (1) be detected in an ethical judgment paradigm involving a simple choice between two options — to treat or not — and (2) varied across seven different cognitive domains that are frequently affected in clinical syndromes. We hypothesized that individuals would weigh risks and benefits differently. More specifically, given evidence that individuals tend to be risk-averse in various contexts (Chauvin, 2018; Kahneman and Tversky, 1979; Thaler et al., 1997), we hypothesized that the public’s choices would be more sensitive to the risks of potential cognitive losses than benefits of potential cognitive gains. Furthermore, we predicted that participants’ risk and benefit sensitivity would vary by cognitive domain, with greater sensitivity to cognitive functions more central to personal identity, such as mood, self-control, and long-term memory (Medaglia et al., 2019a). Specifically, we expected that participants would be more risk and benefit-sensitive to treatments involving deficits in mood and self-control, as these functions tend to be regarded as more central to their personhood (Riis et al., 2008).

## Methods

### Overview

Before completing the primary study, we conducted initial pilot studies of our ethical judgment task to ensure that participants understood the prompts and could respond adequately to test our hypotheses (see “Initial piloting and task design” below). After piloting, we randomly assigned 616 individuals to one of 7 different cognitive conditions: attention (concentration), long-term memory, shor-term (working) memory, mood, language (spoken), self-control, and motor function. In each condition, participants were asked to complete the computerized neuroethical judgment task, then respond to a demographic questionnaire and surveys that assessed their personality (Soto and John, 2017), political ideologies (Inbar et al., 2009), values (Lindeman and Verkasalo, 2005), empathy (Davis, 1983), numeracy (Cokely et al., 2012; Fagerlin et al., 2007), moral foundations (Graham et al., 2009), and moral commitments (Lombrozo, 2009). The sample characteristics and specific procedures are described below. This study was approved by the Institutional Review Board at Drexel University and all participants provided informed consent prior to participating in the study.

### Data and Code Availability

All materials, the data, and R scripts required to reproduce the analyses reported in this paper are available at https://osf.io/n3s9p.

### Participants

A total of 610 participants were recruited from Amazon Mechanical Turk (MTurk). MTurk allows researchers to collect high-quality data rapidly among demographically diverse individuals (Buhrmester et al., 2011). Prior work has shown that MTurk can be reliably used to make inferences about a number of broader populations of interest (Coppock, 2019; Huff and Tingley, 2015; Johnson and Ryan, 2020). The participants were required to be located within the United States, over the age of 18, and fluent in English. Although limitations apply to MTurk studies, studies have shown that the quality of MTurk responses can improve with *a priori* researcher constraints (Cheung et al., 2017; Peer et al., 2014; Thomas and Clifford, 2017). Consistent with these findings, participants were also required to have previously completed at least 100 tasks and maintain at least a 95% requester approval rating on MTurk. Additional exclusions were applied to further ensure that we analyzed high-quality behavioral data (see Exclusions). The final sample included 425 participants (177 females), with a mean age *M* = 36.4 years of age (*SD* = 11). For a full breakdown of demographics, see Table 1. The participants completed the task in an average of *M* = 7.79 min (*SD* = 10.04) and surveys in an average of *M* = 19.27 min (*SD* = 13.15).

**Table 1:** Demographics

Characteristic	*N* = 425
Sex	
Female	177 (42%)
Male	245 (58%)
Other	3 (0.7%)
Age	33 (28, 43)
Race/Ethnicity	
American Indian/Alaska Native	8 (1.9%)
Asian	44 (10%)
Black	41 (9.6%)
Hispanic or Latino	23 (5.4%)
Mixed	12 (2.8%)
Native Hawaiian/Pacific Islander	1 (0.2%)
Other	1 (0.2%)
Prefer not to say	2 (0.5%)
White	293 (69%)
Education	
Undergraduate	242 (57%)
Postgraduate	68 (16%)
High School	114 (27%)
Less than high school	1 (0.2%)
Income	44,000 (26,500, 68,500)
Statistics presented, *n* (%); median (IQR)	

#### Exclusions

Participants were excluded according to participant quality control and data analytic criteria prior to our primary analyses. A total of 191 participants were excluded, yielding a final sample size of 425. Our overall aggressive exclusion rate of 31% is in line with studies of quality control measures on MTurk (Crump et al., 2013; Downs et al., 2010; Paolacci and Chandler, 2014).

##### Participant Exclusions for Quality Control

In line with recommendations for studies conducted using Amazon Mechanical Turk service, careful instructions, *a priori* exclusion criteria, and multi-method approaches for detecting and excluding careless/invalid responses in online samples were applied to further ensure data quality (Crump et al., 2013; Curran, 2016). Specifically, individual trials were excluded if response times were either less than 250 milliseconds (likely impulsive responses) or above 90 s (likely to be non-responses or inattention to the task/time-outs since there was no time limit to make a decision). Additionally, after we applied single-trial exclusions within each participant, some participants were left with less than half the total amount of trials. Patterns of responses with more than half of the total trials removed were likely to be due to consistent impulsive responses or computer-assisted automated completion. Thus, participants were excluded if, after single trial exclusions, they failed to retain at least 40 out of the total of 75 trials. Finally, participants were excluded for failure to pass any of four attention check items randomly administered throughout the survey or a total survey completion time below five minutes, which we found to be implausibly fast to provide valid responses in piloting.

##### Data Analytic Exclusions

Participants were also excluded for selecting a “yes” or “no” response on at least 90% of the total number of trials. Such patterns of stereotyped responses precluded the ability to estimate reliable coefficients in our statistical models. Additionally, these responses might more likely result from individuals not engaging with the information presented on screen during the task. Our analyses yield quantitatively similar results when a less conservative set of exclusions were applied (i.e., excluding only repeat entries or subject entry errors identified when merging survey and task data).

### Experimental Design

#### Stimuli and Procedure

We used a two-alternative forced-choice task to measure individuals’ sensitivity to risks and benefits when serving as a physician hypothetically deciding whether to recommend a treatment for cognitive dysfunction.

Choice tasks are a useful tool in health decision science to measure preferences in which participants are presented with two or more options (Soekhai et al., 2019). Participants then choose which one they prefer. Each option can include attributes that researchers manipulate. Here, we used a discrete two-alternative forced-choice task because, in the real world, clinicians often make the dichotomous choice to recommend a particular treatment or not.

We created seven versions of this task to manipulate the cognitive domain in which participants were asked to make treatment recommendations. Each participant was assigned to one of seven conditions, one for each cognitive domain being assessed: attention/concentration, long-term memory, short-term (working) memory, mood, language (spoken), self-control, and motor function. For a description of each cognitive domain, see Table 2. Participants were randomly assigned to respond only to items containing a single cognitive function so that we could examine the within-subject effects of risk & benefit and the between-subject effect of cognitive condition on choices. For instance, if a participant was assigned to the “attention” condition, they only chose whether to recommend the treatment for attention at different benefit and risk levels.

**Table 2:** Descriptions of cognitive functions and dysfunction

Function	Description	Symptoms of dysfunction
Attention (concentration)	The ability to sustain and switch focus as needed to complete tasks	Has some trouble maintaining and switching focus
Mood	An emotional state, such as feeling sad or happy	Has some trouble feeling happy
Long-term memory	The ability to hold and recall information about the past	Has some trouble remembering people, places, and things
Short-term (“working”) memory	The ability to hold and work with information in mind right now	Has some trouble remembering what they just heard or saw
Language (spoken)	The ability to communicate by speaking	Has some trouble speaking
Motor function	The ability to control one’s own body as intended	Has some trouble moving their body
Self-control	The ability to set and achieve goals by inhibiting unwanted and engaging in wanted behaviors	Has some trouble controlling their thoughts and emotions

Participants were told that they were to take the role of a physician who needed to make treatment decisions with varying potential outcomes for a patient with a cognitive deficit. The mechanism for the treatment of interest was not specified to ensure that the effect of the cognitive domain would not depend on the specifics of the treatment. Before starting the experiment, participants were instructed as follows:

“We are interested in how you think about trade-offs between risk and benefits when treating cognitive deficits. We will ask you to consider a few scenarios. You will need to think about whether you would choose to use a treatment on a patient or not. In the real world, different treatments are associated with different chances of risk and benefits. Your task is to indicate whether or not you would recommend one of the treatments based on the chances that it will improve or harm the stated function all else being equal.”

After reading the instructional slides, participants were shown an example slide to familiarize themselves with the main task. Then, in a sequence of 75 trials, participants were presented with scenarios and they were asked to choose between two treatment options: no treatment or a single treatment. At the start of each trial, a fixation cross was displayed for 200 ms on the center of the screen. After fixation, participants were prompted with the cognitive function and the associated dysfunction symptom for 1000 ms. The cognitive symptom reminder remained on the screen during the entire duration of the trial.

**Fig. 1:**
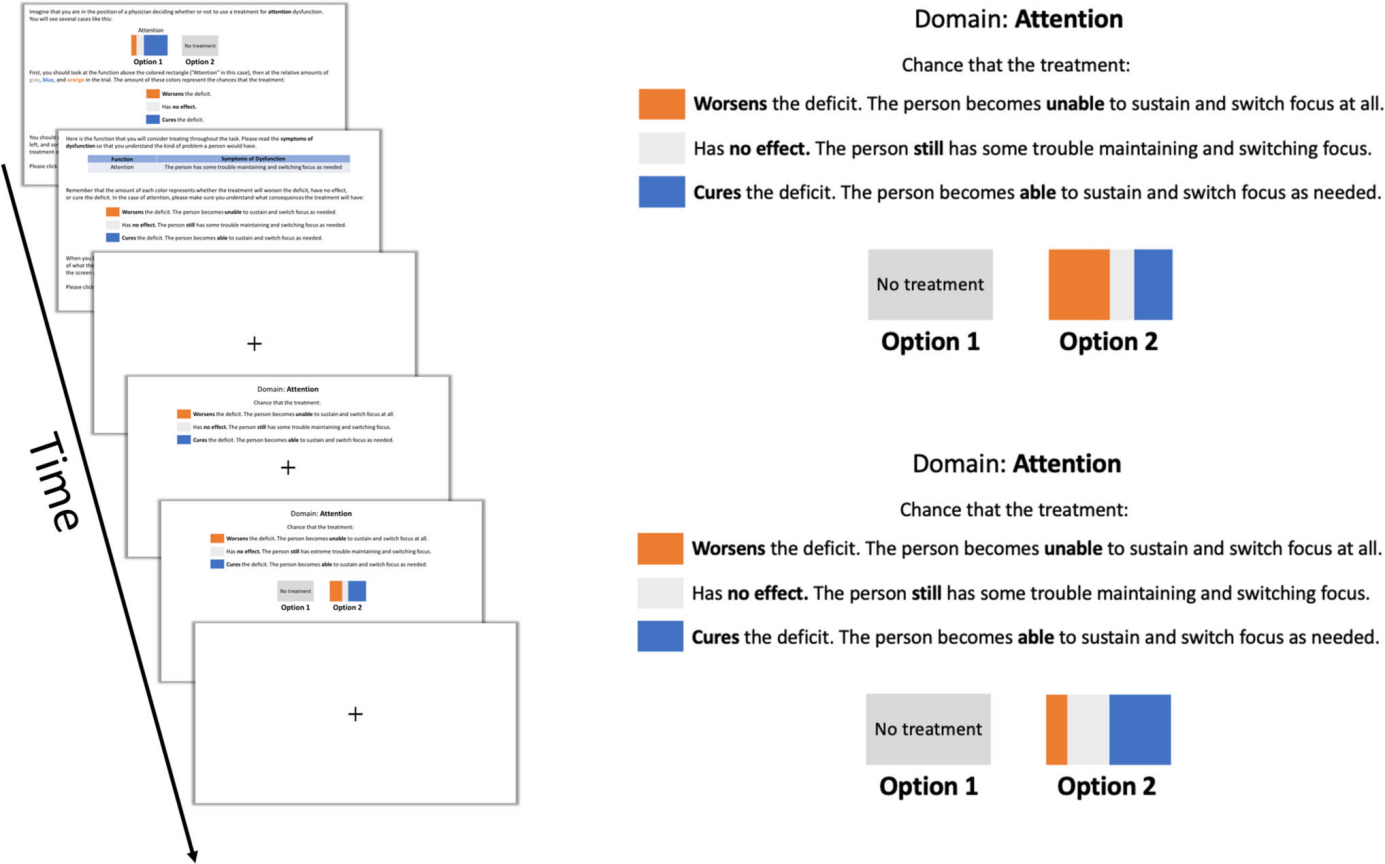
On the left is presented the order of the initial task instructions and a single trial fixation, a reminder of the color mapping, choice stimulus, and inter-trial fixation. On the right are two example trials with different levels of risks and benefits. We used visual analog stimuli to represent the likelihood of treatment effects in stimuli where the sum of the orange, gray, and blue always comprised 100% of the rectangle. In this example, if the participant chose the experimental treatment, she would be recommending one with relatively more risk due to the probability of worsening the deficit being higher, whereas, in the next trial, she would be recommending a treatment with relatively more benefits

While the reminder prompt remained on the screen, participants were presented with an experimental treatment with a varying amount of risk, benefit, or no effect. In each trial, the experimental treatment appeared on the screen in the form of a visual analog painted partly orange, blue, and gray to ensure colorblindness compatibility (Cravit, 2019). Participants were instructed that (1) the amount of orange in the box was proportional to the probability that the cognitive deficit doubled in severity, (2) the amount of gray was proportional to the probability that the treatment has no effect, (3) and the amount of blue was proportional to the probability that the cognitive deficit was removed (see Fig. 1). The no effect (gray) component allowed us to evaluate the influence of risks and benefits independently across trials because they were free to vary quasi-independently (i.e., the orange, gray, and blue together always summed to 100%, but the amount of orange was free to vary from the amount of blue).

The probability of a treatment outcome stated above was directly proportional to the size of the orange and blue regions of the box. The risk and benefit attributes were independently manipulated, with benefit and no effect amounts ranging from 5 to 90% in 5% increments and risk amounts ranging from 5 to 70% in 5% increments, for a total of 75 trials. Participants responded by pressing either “z” for the option on the left or “m” for the option on the right. Upon a response, trials advanced automatically. The left-right orientation of treatment and no treatment stimuli were counterbalanced across trials. See Fig. 1 for a summary of the task stimuli.

#### Initial Piloting and Task Design

Before implementing the primary choice task described above, we tested several paradigms to identify a design that would yield high-quality data for an online study, maintain ecological validity, and be completed in a timely manner. One initial concern when developing a task to emulate a simplified yet realistic trade-off was ensuring ecological validity. In our specific task, we focus on instances where a treatment intended to improve a function may not only have no effect but could potentially worsen other cognitive functions. We chose to limit our design and scope to this single aspect to concentrate on cases where techniques produce isolated effects including transient decrements on a targeted function. These effects can be observed in cases such as intentional “virtual lesions” or other parameters of TMS applied during cognitive processes (Giustiniani et al., 2022; Luber and Lisanby, 2014; Walsh and Pascual-Leone, 2003). While our task is designed to consider scenarios in which treatments aimed at improving a cognitive function have isolated effects or none at all, the larger context of side effects — especially those affecting functions unrelated to the targeted function — holds significant ecological relevance. For instance, even in treatment recommendations, the trade-offs involved in altering cognitive functions are not always directly related to the specific function being targeted (Giustiniani et al., 2022; Rossi et al., 2021), which contrasts with the structure we chose for our design. Future adaptations of the choice task could take these broader across-function trade-off scenarios into account. Having established the scope of our choice task to consider a trade-off directly related to the cognitive function at hand, we first piloted a version of the task with 50 online participants and received low-quality data (e.g., random responding, misunderstanding the mapping between colors, risks, and rewards). To address this, we added persistent reminders of the color mappings, which remained visible on each trial (see Fig. 1). During piloting, we found that 43% of participants showed no statistically significant responsiveness to risk and benefit. Among those who did, risk sensitivity notably diminished beyond a 70% threshold. To optimize task design, we considered restricting the range to focus on risks up to 70%. This design choice, capping risks at 70%, increased the frequency of “yes” responses, allowing for a more reliable estimation of risk sensitivity coefficients. Additionally, this approach retained the capacity to identify participants who showed no statistically significant risk response; in the pilot data, flat responders remained unresponsive across the entire range of risks. A subsequent pilot, with risk levels capped at 70%, confirmed that participants continued to reduce endorsement of treatments well before reaching this threshold. Together, the final task design used up to a 90% chance of benefits and a 70% chance of risks, enabling us to statistically model the effects of each. We ensured that the language describing the tasks and cognitive functions of interest was well understood by participants, that the task could be completed quickly without significant fatigue or disengagement, and that we obtained high-quality data from which to estimate risk and benefit sensitivity for most participants.

#### Demographics

After the judgment task, participants reported their sex, age, race/ethnicity, education, income, field of study, employment status, and political party. In addition, using Likert scales, participants reported their religious upbringing, current religious tradition, degree of religiosity, degree of spirituality, church or other religious meeting attendance, time spent in religious/spiritual activities, relationship status, whether they thought human minds are more like computers or souls, and whether they had ever used a device to optimize cognition before. For details of all the possible responses to the demographic categories and how each item was measured see OSF materials. In this study, we focused only on the results of the judgment task. For a complete list of measures not included in the current report please see our OSF repository https://tinyurl.com/2p8mwpwn.

### Statistical Analyses

All statistical analyses were carried out using R programming language version 4.0.2. Mixed effect modeling were carried out using the *lme4* package (version 1.1-23) & *lmerTest* package (version 3.1-2) (Bates et al., 2015; Kuznetsova et al., 2017). Visualizations were carried out using the *ggplot2* package (Wickham, 2009).

Behavioral choice data were analyzed using general linear mixed-effect modeling with a logit link function. This technique is used to model binary outcome variables, in which the log odds of the outcomes are modeled as a linear combination of the predictor variables that contain both fixed and random effects. Then, within the mixed effect logistic regression, we modeled whether or not participants would recommend one of the treatments as a binary dependent variable, where 1 indicated recommending an experimental treatment and 0 indicated recommending no treatment, and the probability of worsening the defect (amount of orange: risk), and the probability of curing the deficit (amount of blue: benefit) as independent variables. We included a random intercept and slope for risks and benefits across participants. Models included maximal random-effect structures that allowed the model to converge (Barr et al., 2013).

To evaluate individual risk and benefits sensitivity, from the model we extracted the risk and benefit parameters predictions for each individual by using empirical Bayes linear unbiased predictors (EBLUP) for each participant (Henderson, 1975). In practice, because random-effects parameters are unknown, the parameters are estimated and plugged into the predictor, leading to the EBLUP. This method allowed us to estimate variance components for the risks and benefits terms using the estimated random effects for each participant. EBLUPs have been used as indicators of individual differences in the dynamic processes underlying psychological phenomena of interest. For example, clinical psychology has used random effects to quantify individuals’ emotional reactivity (Mroczek et al., 2015). To test if participants were more sensitive to losses than gains, a paired samples *t*-test was used to compare the participant-specific risk and benefit sensitivity estimates. Because we are interested in the difference in magnitude between risk and benefit irrespective of the direction of the effect, a sign inversion was used to ensure mean differences were not driven by the direction of the risk or benefit terms. Then, to test the effect of a cognitive domain on the difference in risk and benefit sensitivity we conducted two separate one-way ANOVAs; one each for risks and benefits.

Models were examined graphically and analytically using a simulation-based approach for assessing the residual diagnostics for multi-level models (Hartig and Lohse, 2021), and assumptions were adequately met. The alpha level for statistical significance was 0.05.

**Fig. 2:**
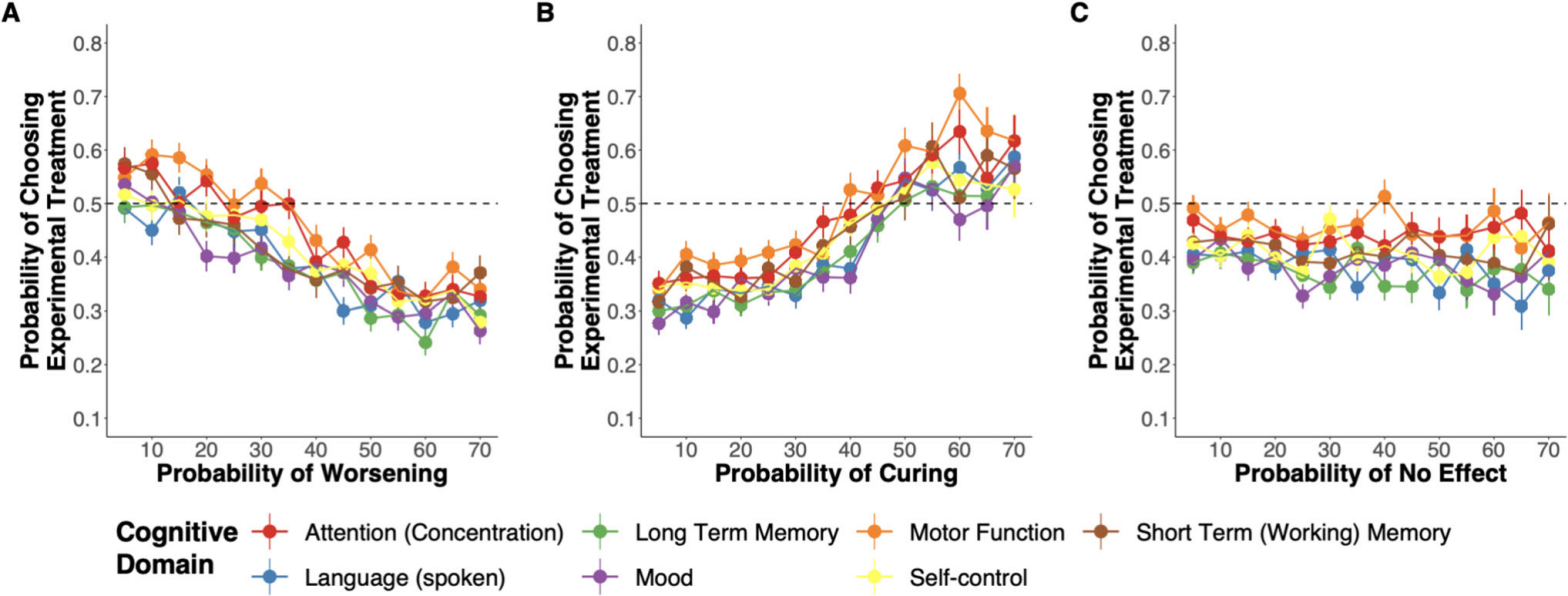
Experimental treatment choice results across each cognitive function domain. **A** Choice proportion for the experimental treatment as a function of the probability of worsening the deficit. **B** Choice proportion for the experimental treatment as a function of the probability of curing the deficit. **C** Choice proportion for the experimental treatment as a function of the probability of no effect on the deficit

## Results

### Descriptive Results

We obtained several descriptive statistics to first examine the behavioral data. On average, participants did not recommend the treatments presented. The probability that the no treatment option was selected across participants was 58.7% (*SD* = 0.17), with 74.6% of participants being more likely to select the no treatment option rather than recommend the experimental treatment. These probabilities are larger than 50%, which is the rate we would expect if choices were made by chance (*t* (424) = 10.41, $p<$ 0.001 when compared to 50% using a one-sample *t*-test).

For each trial, the average trial completion time for the task was *M* = 1.77 s (*SD* = 3.31). Participants did not respond significantly faster to reject than accept the treatment: the average decision to reject the treatment took 1.72 s, whereas the average decision to recommend the treatment took 1.83 s (difference = $-$0.0072 s, 95% CI [$-$0.112, 0.0977], *t*(424) = $-$0135, *p* = 0.893).

### Main Effect of Risk and Benefit Sensitivity

**Fig. 3:**
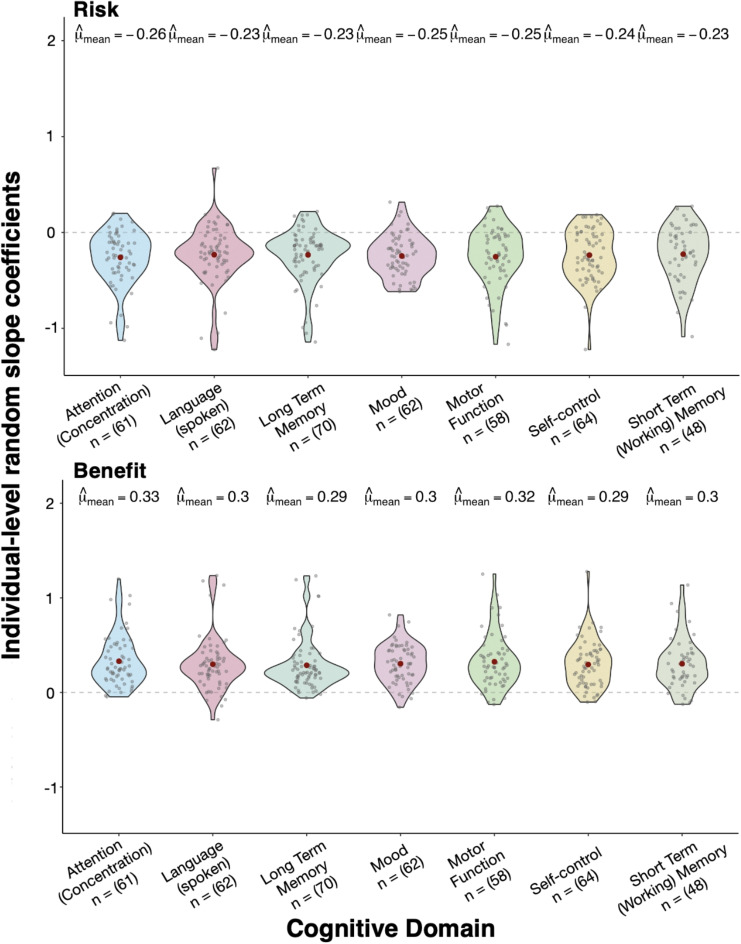
Inter-domain stability. Participant-specific random slopes for risks and benefits across each of the seven cognitive domains assessed in our study. Each violin plot shows the distribution of individual-level coefficients. Red dots represent the mean coefficient ($\hat{\mu }_{\text {mean}}$) for each domain, while small gray points show individual participant values. Sample sizes for each cognitive domain are displayed below the domain labels. The top panel shows risk sensitivity coefficients, which are predominantly negative across all domains, while the bottom panel displays benefit sensitivity coefficients, which are consistently positive. Notably, the pattern across cognitive domains was consistent, supporting the idea that participants evaluate risks versus benefits similarly across different cognitive domains

As expected, participants generally chose in line with not recommending treatments with a higher probability of worsening the deficit (see Fig. 2A) and, recommending treatments with a higher probability of curing the deficit (Fig. 3B). The odds of choosing to recommend the treatment decreased as the probability of worsening the deficit increased (OR = 0.79, 95% CI [0.75, 0.82], $p<$ 0.001). Additionally, we also found that the odds of choosing to recommend the treatment increased as the probability of curing the deficit increased (OR = 1.35, 95% CI [1.3, 1.41], $p<$ 0.001).

### Cognitive Domains

We did not find that the domain of deficit influenced people’s treatment recommendations. Table 3 shows the average predicted participant level change in the log odds of choosing the experimental treatment for both risk and benefit across each cognitive domain. The ANOVA did not show support for any effect of the cognitive domain ($F_{6, 418}$ = 0.11, *p* = 0.995). Likewise, the effect of the cognitive domain for benefit was also not significant, ($F_{6, 418}$ = 0.23, *p* =.97) thus indicating inter-domain stability across the cognitive domains considered within our study.

**Table 3:** Average predicted participants level change in the log odds for risk and benefit for each cognitive domain

Domain	eBLUP$_{risk}$	SD$_{risk}$	eBLUP$_{benefit}$	SD$_{benefit}$
Attention (concentration)	$-$0.258	0.278	0.327	0.267
Language (spoken)	$-$0.231	0.302	0.295	0.287
Long-term memory	$-$0.233	0.279	0.284	0.262
Mood	$-$0.245	0.217	0.301	0.202
Motor function	$-$0.252	0.306	0.322	0.282
Self-control	$-$0.235	0.268	0.292	0.246
Short-term (working) memory	$-$0.225	0.296	0.299	0.268

*Note:* *eBLUP* empirical best linear unbiased prediction, *SD* standard deviation

### Influence of Risks and Benefits

**Fig. 4:**
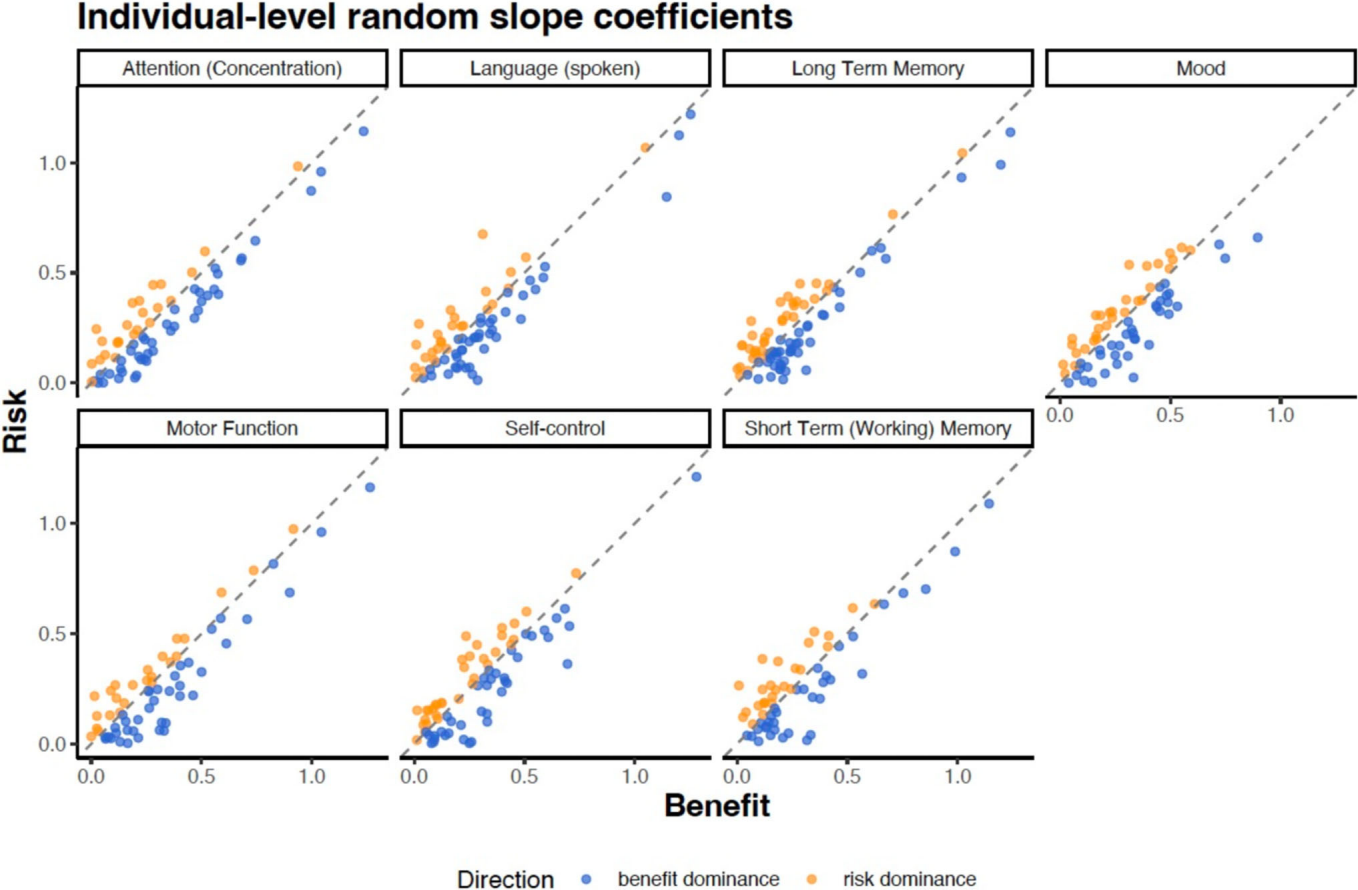
Benefit dominance across cognitive functions. Points represent the absolute value of the participant random slope estimates along the identity line for each cognitive function. The color indicates where the point was above or below the identity. If a participant is above the identity, they were more sensitive to risks; below the identity indicates more sensitivity to benefits

While we expected that people would weigh the probability of a decline in cognitive function more than the probability of benefits, the paired samples *t*-test revealed that the difference in absolute value of EBLUPs between benefit and risk ($M_{diff}$ = $-$0.035) was significant (difference = 95% CI [$-$0.045, $-$0.025], *t*(424) = $-$7.21, $p<$.001, Cohen’s *d* = $-$0.35, CI [$-$0.45, $-$0.25]) with a slightly higher weight placed on benefits (*M* = 0.32, *SD* = 0.25) compared to risks (*M* = 0.28, *SD* = 0.24). To illustrate this benefit dominance, we replotted the absolute values of the individual-level coefficients shown in Fig. 3 for each cognitive domain. If participants weighted risks and benefits equally, we would expect all data points to fall on the identity line. Consistently, across each domain, we observed that participants tend to have unequal weighting, with evidence indicating that a majority are benefit-dominant (see Fig. 4).

### Exploratory Analysis of Response Times

We conducted an additional exploratory analysis of response times. When making ethical judgments, how quickly someone responds may indicate how they arrived at their judgment (Baron and Gürçay, 2017). For example, longer response times may suggest that participants grappled with more decision conflict while deliberating, while shorter responses may suggest that they arrived at their judgments more easily (Konovalov and Krajbich, 2019; Krajbich et al., 2015). Therefore, when making ethical judgments, response times may provide valuable insights into the strength-of-preferences participants may have for one treatment over another. We fitted a linear mixed model to predict log response times as a function of the cognitive domain, risk, and benefit sensitivity separately for trials where participants did and did not select the experimental treatment. The model included subject-level random intercepts and slopes for risk and benefits. As in previous research (e.g., Konovalov & Krajbich, 2019), participants generally took longer on more difficult decisions. For trials where participants selected the treatment, the mixed-effects regression indicates that people choose faster as the probability of curing the deficit increased beta = $-$0.03, 95% CI [$-$0.06, $-$0.01], *p* = 0.003. However, they were not similarly sensitive to the probability of worsening the deficit (beta = $-$0.02, 95% CI [$-$0.04, 0.00231], *p* = 0.080). For trials where participants selected the no treatment option, we found the opposite: people chose no treatment faster as the probability of worsening the deficit increased (beta = $-$0.03, 95% CI [$-$0.05, $-$0.01], $p<$.001) though they were not similarly sensitive to the probability of curing the deficit (beta = $-$0.00859, 95% CI [$-$0.03, 0.01], *p* = 0.377). Further investigation of these results is reported in another study.

## Discussion

In a two-alternative forced-choice experiment measuring risk and benefit sensitivity in the public when recommending hypothetical treatments for cognitive deficits, we found partial support for our hypotheses. Consistent with our hypothesis, participants weighted risk and benefits differently. However, contrary to our hypothesis, participants were more sensitive to benefits than to risks. Participants also showed unexpected inter-domain stability across cognitive dysfunctions.

Typically, in monetary risky decision-making, most people are expected to be sensitive to risks — they tend to hold on to what they have instead of pursuing more (Kahneman and Tversky, 1979; Thaler et al., 1997). Therefore, the most surprising finding — that individuals were more sensitive to probabilities of benefit than risks to treat others — bears special consideration. One possible explanation draws from a prominent theory of decision-making under risk: *prospect theory*. Originally developed in the context of monetary decisions, prospect theory is a descriptive theory of how people make decisions under risk and uncertainty (Kahneman and Tversky, 1979). Near the turn of the 21st century, Treadwell & Lenert noted that prospect theory could conceptually account for the differential weighting of short-term and long-term treatment outcomes, the influences of choice framing, and judgments involving the chances of patient survival (Treadwell and Lenert, 1999). Since that time, prospect theory has been adapted to account for a variety of medical decisions (Phillips et al., 2011; Rasiel et al., 2005; Schwartz et al., 2008; van Osch et al., 2006; Winter and Parker, 2007). While prospect theory has been successfully applied in other treatment domains, there have not been thorough applications of prospect theory to study ethical choices about cognitive treatments (for discussion see, Fernandez et al. 2022).

In the context of prospect theory, it is possible that people make choices with respect to a reference point — a status quo starting position along a dimension of value (Kahneman and Tversky, 1979). For treatment recommendations, a reference point could be set along the healthiness of a given cognitive state. Prospect theory predicts that people tend to be risk-seeking below the reference point and risk-averse above it. In our experiment, if participants used healthy cognition as the reference point, they would be expected to weigh an improvement in a deficit more heavily than an equivalent decrement in that deficit, consistent with our finding that participants systematically weigh the prospects of curing a deficit more than expected. However, it is worth noting that an alternative explanation is that participants may assess positive outcomes differently from negative outcomes, displaying asymmetric sensitivity to changes in magnitude for these outcomes. While we designed the vignettes to maximize clarity of the symptoms and benefits of treatment, they may have made it more likely for participants to endorse treatments than respond to risks overall. In the context of our current experiment, participants may not have perceived a unit change in worsening the deficit as equivalent to a unit change in curing the deficit. This cannot be ruled out with the current data because we did not explicitly manipulate whether the worsening condition seemed as equally negative to participants as the cure condition seemed positive. Manipulating the relative value of each end of the judgments could be informative. For instance, more severe symptom levels or less guaranteed curative effects could make participants more hesitant to select the treatments.

We did not detect any statistically significant differences in risk and benefit sensitivity across domains of dysfunction. Given that we applied equal manipulations of the probability of risks and benefits across domains, participants appeared to value the different domains of dysfunction similarly. There is reason to suspect that insensitivity to domain may reflect the public’s impartiality about which particular cognitive domains are targeted by cognitive repair (Cabrera et al., 2015b). Weighing the consequences of recommending the treatment is fundamentally a choice about whether or not to help an individual or not. As our data suggest, the public may be more concerned with how likely treatments are to benefit rather than harm a patient. Alternatively, it is possible that the simplified descriptions of the deficits were not sufficiently detailed or salient to evoke differences in choices. For instance, vignette studies of medical judgments show that participants are sensitive to peripheral threat cues like the invasiveness of the treatment (Gong et al., 2013; Medaglia et al., 2019b), or perceived susceptibility to the deficit (Gallagher et al., 2011). To further test the inter-domain stability suggested here, future work could consider stronger manipulations such as using representative images rather than descriptions for each domain of dysfunction (Dan, 2017; Powell et al., 2015).

We also found that participants exhibited faster response times when selecting treatments with a higher probability of curing the deficit, or when selecting no treatment if it had a higher probability of worsening the deficit. Interestingly, response times often decrease with strength-of-preference, as supported by prior research (Konovalov and Krajbich, 2019; Krajbich et al., 2015). Strength-of-preference refers to the extent to which one option is favored over another. For instance, if a person consistently chooses to read a book over watching a movie, their strength-of-preference for reading is high. Similarly, if a participant consistently chooses not to recommend the experimental treatment, their strength-of-preference for no treatment is high. In our study, the differences in response times between treatment recommendations suggest average differences in the strength-of-preference for each treatment. Future studies should explore additional factors that may contribute to response times such as individual differences (Ratcliff and Childers, 2015; Ratcliff et al., 2015; Schubert et al., 2019). For example, researchers could investigate whether individuals with different moral attitudes have differing strengths-of-preference when selecting the treatment option mediated by their values, which could result in faster response times.

Overall, our results suggest when members of the public might be willing to recommend experimental or conventional therapies for cognitive dysfunction. In judgments where the details of the deficits are relatively sparse, choices might be dominated by the weight placed on risks and benefits relative to the domain of treatment. Though we expected the weighting between attributes to be risk-dominated, our results suggest a more equal weighting between risk and benefits, with the probability of benefits weighted slightly larger relative to other factors in treatment decisions. We suspect that these patterns could reflect the public’s preferences toward cognitive repair. This notion aligns with prior survey research showing that the public is more willing to endorse neuromodulation for repair than enhance to cognition, especially when considering its use on others (Medaglia et al., 2019a). Cabrera and colleagues have also found that the public tends to focus on personal responsibility when discussing concerns about and the efficacy of psychiatric treatments, including neuromodulation (Cabrera et al., 2018). Taken together, these findings suggest that the public places an emphasis on helping others with cognitive dysfunction, weighing the benefits of treatments equally to or slightly more heavily than the potential harms.

### Limitations and Future Directions

It is worth noting some limitations of this study. First, participants were asked to respond as if they were physicians making hypothetical treatment recommendations. However, most of our participants were not physicians. Prior research has shown that participant responses to hypotheticals can differ from how they would act in that real situation (Chang et al., 2009). Future studies should investigate how risk and benefit sensitivity influence real-world choices. By doing so, we can determine whether our task-based measures of risk and benefit sensitivity mediate public opinions, clinician recommendations, and patient preferences about treatments for cognitive dysfunction. We also recommend future research test whether risk and benefit sensitivity varies for decisions made on behalf of oneself rather than others (Arrieta et al., 2017; Garcia-Retamero and Galesic, 2012; Haslam et al., 2020; Medaglia et al., 2019a; Ubel et al., 2011). Examining choices with ambiguity is an important future direction in light of known effects in other decision paradigms and given their ecological validity (see e.g., Attema et al. 2018). Moreover, given that risk and benefit sensitivity can be influenced by cognitive or mood states such as depression, additional research in clinical contexts is warranted (Bayer et al., 2019). The influence of sociocultural, religious, moral, and ideological affiliations on risk and benefit sensitivity could also be examined. Finally, while prior research suggests that well-screened survey data collected via Mechanical Turk can be representative of data acquired in a person (Buhrmester et al., 2011), future in-person studies could test for generalization.

### Conclusion

Individuals’ risk and benefit sensitivity may be related to judgments involving cognitive treatments that depend on context-specific mediating factors. This insight broadens the scope of studies on risky decision-making in ethical settings and may have real-world implications in settings where the public are stakeholder in consent to research or clinical procedures. Our work also presents a novel quantitative neuroethics approach for measuring risk and benefit sensitivity when modifying cognitive functions that could inform tensions in informed consent and risk communication. By using quantitative strategies to study judgments and preferences about cognitive treatments, we can begin to develop a mature understanding of ethical judgments regarding the use of technologies to repair and enhance various cognitive functions, with the aim of informing public and professional policy, education, and biomedical interventions.

## Data Availability

Materials and raw data can be accessed at https://osf.io/n3s9p

## Footnotes

1 The definition of risk continues to be a debated topic in applied neuroethics. Here, we use the term in a general sense, referring to potential negative outcomes. This usage differs from some definitions found in economics and decision-making literature, where risk can denote variance over potential outcomes. Risk has also been referred to as undesirable events, danger, or losses (Aven, 2012; Frey et al., 2017).

## References

[CR1] Amadio, J., Bi, G.-Q., Boshears, P. F., Carter, A., Devor, A., Doya, K., . . . Singh, I. (2018). Neuroethics questions to guide ethical research in the international brain initiatives. *Neuron,**100*(1), 19–36. 10.1016/j.neuron.2018.09.021 10.1016/j.neuron.2018.09.021 30308169

[CR2] Arrieta, A., García-Prado, A., González, P., & Pinto-Prades, J. L. (2017). Risk attitudes in medical decisions for others: An experimental approach. *Health Economics,**26*(Suppl 3), 97–113. 10.1002/hec.3628 29285873 10.1002/hec.3628

[CR3] Attema, A. E., Bleichrodt, H., & L’Haridon, O. (2018). Ambiguity preferences for health. *Health Economics,**27*(11), 1699–1716. 10.1002/hec.3795 29971896 10.1002/hec.3795 PMC6221042

[CR4] Aven, T. (2012). The risk concept–historical and recent development trends. *Reliability Engineering & System Safety,**99*, 33–44.

[CR5] Banjo, O. C., Nadler, R., & Reiner, P. B. (2010). Physician attitudes towards pharmacological cognitive enhancement: Safety concerns are paramount. *PLOS ONE,**5*(12), e14322. 10.1371/journal.pone.0014322 21179461 10.1371/journal.pone.0014322 PMC3001858

[CR6] Baron, J., & Gürçay, B. (2017). A meta-analysis of response-time tests of the sequential two-systems model of moral judgment. *Memory & Cognition,**45*(4), 566–575. 10.3758/s13421-016-0686-8 28028781 10.3758/s13421-016-0686-8

[CR7] Barr, D. J., Levy, R., Scheepers, C., & Tily, H. J. (2013). Random effects structure for confirmatory hypothesis testing: Keep it maximal. *Journal of Memory and Language,**68*(3). 10.1016/j.jml.2012.11.001 10.1016/j.jml.2012.11.001 PMC3881361 24403724

[CR8] Barrett, M. S., Chua, W.-J., Crits-Christoph, P., Gibbons, M. B., Casiano, D., & Thompson, D. (2008). Early withdrawal from mental health treatment: Implications for psychotherapy practice. *Psychotherapy (Chicago, Ill),**45*(2), 247–267. 19838318 10.1037/0033-3204.45.2.247 PMC2762228

[CR9] Bates, D., Mächler, M., Bolker, B., & Walker, S. (2015). Fitting linear mixed-effects models using lme4. *Journal of Statistical Software,**67*(1). 10.18637/jss.v067.i01

[CR10] Bayer, Y. M., Shtudiner, Z., Suhorukov, O., & Grisaru, N. (2019). Time and risk preferences, and consumption decisions of patients with clinical depression. *Journal of Behavioral and Experimental Economics,**78*, 138–145. 10.1016/j.socec.2019.01.003

[CR11] Bell, S., Partridge, B., Lucke, J., & Hall, W. (2013). Australian university students’ attitudes towards the acceptability and regulation of pharmaceuticals to improve academic performance. *Neuroethics,**6*(1), 197–205. 10.1007/s12152-012-9153-9

[CR12] Buhrmester, M., Kwang, T., & Gosling, S. D. (2011). Amazon’s Mechanical Turk: A new source of inexpensive, yet high-quality, data? *Perspectives on Psychological Science: A Journal of the Association for Psychological Science,**6*(1), 3–5. 10.1177/1745691610393980 26162106 10.1177/1745691610393980

[CR13] Cabrera, L. Y., Brandt, M., McKenzie, R., & Bluhm, R. (2018). Comparison of philosophical concerns between professionals and the public regarding two psychiatric treatments. *AJOB Empirical Bioethics,**9*(4), 252–266. 10.1080/23294515.2018.1512534 30398397 10.1080/23294515.2018.1512534

[CR14] Cabrera, L. Y., Evans, E. L., & Hamilton, R. H. (2014). Ethics of the electrified mind: Defining issues and perspectives on the principled use of brain stimulation in medical research and clinical care. *Brain Topography,**27*(1), 33–45. 10.1007/s10548-013-0296-8 23733209 10.1007/s10548-013-0296-8 PMC3806889

[CR15] Cabrera, L. Y., Fitz, N. S., & Reiner, P. B. (2015a). Empirical support for the moral salience of the therapy-enhancement distinction in the debate over cognitive, affective and social enhancement. *Neuroethics,**8*(3), 243–256. 10.1007/s12152-014-9223-2

[CR16] Cabrera, L. Y., Fitz, N. S., & Reiner, P. B. (2015). Reasons for comfort and discomfort with pharmacological enhancement of cognitive, affective, and social domains. *Neuroethics,**8*(2), 93–106. 10.1007/s12152-014-9222-3

[CR17] Chang, J. B., Lusk, J. L., & Norwood, F. B. (2009). How closely do hypothetical surveys and laboratory experiments predict field behavior? *American Journal of Agricultural Economics,**91*(2), 518–534. 10.1111/j.1467-8276.2008.01242.x

[CR18] Chauvin, B. (2018). Individual differences in the judgment of risks: Sociodemographic characteristics, cultural orientation, and level of expertise. In M. Raue, E. Lermer, & B. Streicher (Eds.), *Psychological perspectives on risk and risk analysis: Theory, models, and applications* (pp. 37–61). Cham: Springer International Publishing.

[CR19] Cheung, J. H., Burns, D. K., Sinclair, R. R., & Sliter, M. (2017). Amazon Mechanical Turk in organizational psychology: An evaluation and practical recommendations. *Journal of Business and Psychology,**32*(4), 347–361. 10.1007/s10869-016-9458-5

[CR20] Cokely, E. T., Galesic, M., Schulz, E., Garcia-Retamero, R., & Ghazal, S. (2012). Measuring risk literacy: The Berlin numeracy test. *Judgment and Decision Making,**7*(1), 23.

[CR21] Conrad, E. C., Humphries, S., & Chatterjee, A. (2019). Attitudes toward cognitive enhancement: The role of metaphor and context. *AJOB Neuroscience,**10*(1), 35–47. 10.1080/21507740.2019.1595771 31070552 10.1080/21507740.2019.1595771

[CR22] Coppock, A. (2019). Generalizing from survey experiments conducted on Mechanical Turk: A replication approach. *Political Science Research and Methods,**7*(3), 613–628. 10.1017/psrm.2018.10

[CR23] Cravit, R. (2019). *How to use color blind friendly palettes to make your charts accessible*. https://venngage.com/blog/color-blind-friendly-palette/

[CR24] Crump, M. J. C., McDonnell, J. V., & Gureckis, T. M. (2013). Evaluating Amazon’s Mechanical Turk as a tool for experimental behavioral research. *PLOS ONE,**8*(3), e57410. 10.1371/journal.pone.0057410 23516406 10.1371/journal.pone.0057410 PMC3596391

[CR25] Curran, P. G. (2016). Methods for the detection of carelessly invalid responses in survey data. *Journal of Experimental Social Psychology,**66*, 4–19. 10.1016/j.jesp.2015.07.006

[CR26] Dan, V. (2017). *Integrative framing analysis: Framing health through words and visuals*. Routledge.

[CR27] Davis, M. H. (1983). Measuring individual differences in empathy: Evidence for a multidimensional approach. *Journal of Personality and Social Psychology,**44*(1), 113–126. 10.1037/0022-3514.44.1.113

[CR28] Dinh, C., Humphries, S., & Chatterjee, A. (2020). *Public opinion on cognitive enhancement varies across different situations*. PsyArXiv. 10.31234/osf.io/ydqru 10.1080/21507740.2020.1811797 33196348

[CR29] Downs, J. S., Holbrook, M. B., Sheng, S., & Cranor, L. F. (2010). Are your participants gaming the system? Screening Mechanical Turk workers. *Proceedings of the SIGCHI conference on human factors in computing systems* (pp. 2399–2402). New York, NY, USA: Association for Computing Machinery.

[CR30] Eichler, H.-G., Bloechl-Daum, B., Brasseur, D., Breckenridge, A., Leufkens, H., Raine, J., . . . Rasi, G. (2013). The risks of risk aversion in drug regulation. *Nature Reviews Drug Discovery,**12*(12), 907–916. 10.1038/nrd4129 10.1038/nrd4129 24232377

[CR31] Ezzyat, Y., Wanda, P. A., Levy, D. F., Kadel, A., Aka, A., Pedisich, I., . . . Kahana, M. J. (2018). Closed-loop stimulation of temporal cortex rescues functional networks and improves memory. *Nature Communications,**9*(1), 365. 10.1038/s41467-017-02753-0 10.1038/s41467-017-02753-0 PMC5802791 29410414

[CR32] Faber, N. S., Douglas, T., Heise, F., & Hewstone, M. (2015). Cognitive enhancement and motivation enhancement: An empirical comparison of intuitive judgments. *AJOB Neuroscience,**6*(1), 18–20. 10.1080/21507740.2014.991847

[CR33] Fagerlin, A., Zikmund-Fisher, B. J., Ubel, P. A., Jankovic, A., Derry, H. A., & Smith, D. M. (2007). Measuring numeracy without a math test: Development of the subjective numeracy scale. *Medical Decision Making: An International Journal of the Society for Medical Decision Making,**27*(5), 672–680. https://doi.org/10.1177/0272989X07304449 17641137 10.1177/0272989X07304449 17641137

[CR34] Fernandez, K., Hamilton, R., Cabrera, L., & Medaglia, J. D. (2022). Context-dependent risk & benefit sensitivity mediate judgments about cognitive enhancement. *AJOB neuroscience,**13*(1), 73–77. 34931943 10.1080/21507740.2021.2001077 PMC9867800

[CR35] Fitz, N. S., Nadler, R., Manogaran, P., Chong, E. W. J., & Reiner, P. B. (2014). Public attitudes toward cognitive enhancement. *Neuroethics,**7*(2), 173–188. 10.1007/s12152-013-9190-z

[CR36] Frey, R., Pedroni, A., Mata, R., Rieskamp, J., & Hertwig, R. (2017). Risk preference shares the psychometric structure of major psychological traits. *Science advances,**3*(10), e1701381. 28983511 10.1126/sciadv.1701381 PMC5627985

[CR37] Funk, C., Kennedy, B., & Sciupac, E. (2016). U.S. Public wary of biomedical technologies to ‘enhance’ human abilities. *Pew Research Center*, *132*.

[CR38] Galizzi, M. M., Miraldo, M., & Stavropoulou, C. (2016). In sickness but not in wealth: Field evidence on patients’ risk preferences in financial and health domains. *Medical Decision Making,**36*(4), 503–517. 10.1177/0272989X15626406 26856889 10.1177/0272989X15626406

[CR39] Gallagher, K. M., Updegraff, J. A., Rothman, A. J., & Sims, L. (2011). Perceived susceptibility to breast cancer moderates the effect of gain-and loss-framed messages on use of screening mammography. *Health psychology: official journal of the Division of Health Psychology, American Psychological Association,**30*(2), 145–152. 10.1037/a0022264 21401248 10.1037/a0022264 PMC4679369

[CR40] Garcia-Retamero, R., & Galesic, M. (2012). Doc, what would you do if you were me? On self–other discrepancies in medical decision making. *Journal of Experimental Psychology: Applied,**18*(1), 38–51. 10.1037/a0026018 22039766 10.1037/a0026018

[CR41] Giustiniani, A., Vallesi, A., Oliveri, M., Tarantino, V., Ambrosini, E., Bortoletto, M., . . . et al. (2022). A questionnaire to collect unintended effects of transcranial magnetic stimulation: A consensus based approach. *Clinical Neurophysiology,**141*, 101–108. 10.1016/j.clinph.2022.06.008 35798667

[CR42] Gong, J., Zhang, Y., Yang, Z., Huang, Y., Feng, J., & Zhang, W. (2013). The framing effect in medical decision-making: A review of the literature. *Psychology, Health & Medicine,**18*(6), 645–653. 10.1080/13548506.2013.766352 10.1080/13548506.2013.766352 23387993

[CR43] Graham, J., Haidt, J., & Nosek, B. A. (2009). Liberals and conservatives rely on different sets of moral foundations. *Journal of Personality and Social Psychology,**96*(5), 1029–1046. 10.1037/a0015141 19379034 10.1037/a0015141

[CR44] Greely, H., Sahakian, B., Harris, J., Kessler, R. C., Gazzaniga, M., Campbell, P., & Farah, M. J. (2008). Towards responsible use of cognitive-enhancing drugs by the healthy. *Nature,**456*(7223), 702–705. 10.1038/456702a 19060880 10.1038/456702a

[CR45] Hartig, F., & Lohse, L. (2021). *DHARMa: Residual diagnostics for hierarchical (multi-level / mixed) regression models.*

[CR46] Haslam, M., Yaden, D. B., & Medaglia, J. D. (2020). Moral framing and mechanisms influence public willingness to optimize cognition. *Journal of Cognitive Enhancement*, 1–12.

[CR47] Hauber, B., Fairchild, A., & Johnson, R. (2013). Quantifying benefit-risk preferences for medical interventions: An overview of a growing empirical literature. *Applied Health Economics and Health Policy*, *11*(4). 10.1007/s40258-013-0028-y 23637054

[CR48] Henderson, C. R. (1975). Best linear unbiased estimation and prediction under a selection model. *Biometrics,**31*(2), 423–447. 10.2307/2529430 1174616

[CR49] Huff, C., & Tingley, D. (2015). “Who are these people?’’ Evaluating the demographic characteristics and political preferences of MTurk survey respondents. *Research & Politics,**2*(3), 2053168015604648. 10.1177/2053168015604648

[CR50] Inbar, Y., Pizarro, D. A., & Bloom, P. (2009). Conservatives are more easily disgusted than liberals. *Cognition and Emotion,**23*(4), 714–725. 10.1080/02699930802110007

[CR51] Johnson, D., & Ryan, J. B. (2020). Amazon Mechanical Turk workers can provide consistent and economically meaningful data. *Southern Economic Journal,**87*(1), 369–385. 10.1002/soej.12451

[CR52] Johnson, L. A., Nebeck, S. D., Muralidharan, A., Johnson, M. D., Baker, K. B., & Vitek, J. L. (2016). Closed-loop deep brain stimulation effects on parkinsonian motor symptoms in a non-human primate - Is beta enough? *Brain Stimulation,**9*(6), 892–893. 10.1016/j.brs.2016.06.051 27401045 10.1016/j.brs.2016.06.051 PMC5143196

[CR53] Kahneman, D., & Tversky, A. (1979). Prospect theory: An analysis of decision under risk. *Econometrica,**47*(2), 263–291. 10.2307/1914185

[CR54] Kim, C. K., Adhikari, A., & Deisseroth, K. (2017). Integration of optogenetics with complementary methodologies in systems neuroscience. *Nature Reviews. Neuroscience,**18*(4), 222–235. 10.1038/nrn.2017.15 28303019 10.1038/nrn.2017.15 PMC5708544

[CR55] Klein, E., Daza, N. M., Dasgupta, I., MacDuffie, K., Schönau, A., Flynn, G., . . . Goering, S. (2023). Views of stakeholders at risk for dementia about deep brain stimulation for cognition. *Brain stimulation,**16*(3), 742–747. 10.1016/j.brs.2023.04.007 PMC10576447 37076043

[CR56] Konovalov, A., & Krajbich, I. (2019). Revealed strength of preference: Inference from response times. *Judgment and Decision Making*, 14.

[CR57] Krajbich, I., Bartling, B., Hare, T., & Fehr, E. (2015). Rethinking fast and slow based on a critique of reaction-time reverse inference. *Nature Communications,**6*(1), 7455. 10.1038/ncomms8455 26135809 10.1038/ncomms8455 PMC4500827

[CR58] Kuznetsova, A., Brockhoff, P. B., & Christensen, R. H. B. (2017). lmerTest Package: Tests in linear mixed effects models. *Journal of Statistical Software,**82*(1), 1–26. 10.18637/jss.v082.i13

[CR59] Li, M., Kim, H., Sereika, S. M., Nissley, T. J., & Lingler, J. H. (2022). Willingness to participate in clinical research among individuals with cognitive impairment. *Research in Gerontological Nursing,**15*(2), 76–84. 35148207 10.3928/19404921-20220131-01 PMC9341492

[CR60] Lindeman, M., & Verkasalo, M. (2005). Measuring values with the short Schwartz’s value survey. *Journal of Personality Assessment,**85*(2), 170–178. 10.1207/s15327752jpa8502_09 16171417 10.1207/s15327752jpa8502_09

[CR61] Lombrozo, T. (2009). The role of moral commitments in moral judgment. *Cognitive Science,**33*(2), 273–28. 10.1111/j.1551-6709.2009.01013.x 21585471 10.1111/j.1551-6709.2009.01013.x

[CR62] Luber, B., & Lisanby, S. H. (2014). Enhancement of human cognitive performance using transcranial magnetic stimulation (TMS). *Neuroimage,**85*, 961–970. 23770409 10.1016/j.neuroimage.2013.06.007 PMC4083569

[CR63] Marrero, R. J., Fumero, A., de Miguel, A., & Peñate, W. (2020). Psychological factors involved in psychopharmacological medication adherence in mental health patients: A systematic review. *Patient Education and Counseling,**103*(10), 2116–2131. 10.1016/j.pec.2020.04.030 32402489 10.1016/j.pec.2020.04.030

[CR64] Medaglia, J. D., Kuersten, A., & Hamilton, R. H. (2020). Protecting decision-making in the era of neuromodulation. *Journal of Cognitive Enhancement,**4*(4), 469–481. 10.1007/s41465-020-00171-7

[CR65] Medaglia, J. D., Yaden, D. B., Helion, C., & Haslam, M. (2019a). Moral attitudes and willingness to enhance and repair cognition with brain stimulation. *Brain Stimulation,**12*(1), 44–53. 10.1016/j.brs.2018.09.014 30309762 10.1016/j.brs.2018.09.014 PMC6685214

[CR66] Medaglia, J. D., Yaden, D. B., Helion, C., & Haslam, M. (2019b). Moral attitudes and willingness to enhance and repair cognition with brain stimulation. *Brain stimulation,**12*(1), 44–53. 30309762 10.1016/j.brs.2018.09.014 PMC6685214

[CR67] Medaglia, J. D., Zurn, P., Sinnott-Armstrong, W., & Bassett, D. S. (2017). Mind control as a guide for the mind. *Nature Human Behaviour,**1*(6), 1–8. 10.1038/s41562-017-0119

[CR68] Mohr, D. C., Ho, J., Duffecy, J., Baron, K. G., Lehman, K. A., Jin, L., & Reifler, D. (2010). Perceived barriers to psychological treatments and their relationship to depression. *Journal of Clinical Psychology,**66*(4), 394–409. 10.1002/jclp.20659 20127795 10.1002/jclp.20659 PMC2907887

[CR69] Mroczek, D. K., Stawski, R. S., Turiano, N. A., Chan, W., Almeida, D. M., Neupert, S. D., & Spiro, A., III. (2015). Emotional reactivity and mortality: Longitudinal findings from the VA normative aging study. *The Journals of Gerontology: Series B,**70*(3), 398–406. 10.1093/geronb/gbt107 10.1093/geronb/gbt107 PMC4542645 24170714

[CR70] Nadler, R. C., & Reiner, P. B. (2010). A call for data to inform discussion on cognitive enhancement. *BioSocieties,**5*(4), 481–482. 10.1057/biosoc.2010.30

[CR71] Paolacci, G., & Chandler, J. (2014). Inside the Turk: Understanding Mechanical Turk as a participant pool. *Current Directions in Psychological Science,**23*(3), 184–188. 10.1177/0963721414531598

[CR72] Partridge, B., Lucke, J., & Hall, W. (2012). A comparison of attitudes toward cognitive enhancement and legalized doping in sport in a community sample of Australian adults. *AJOB Primary Research,**3*(4), 81–86. 10.1080/21507716.2012.720639

[CR73] Peer, E., Vosgerau, J., & Acquisti, A. (2014). Reputation as a sufficient condition for data quality on Amazon Mechanical Turk. *Behavior Research Methods,**46*(4), 1023–1031. 10.3758/s13428-013-0434-y 10.3758/s13428-013-0434-y 24356996

[CR74] Phillips, L. L., Allen, R. S., Harris, G. M., Presnell, A. H., Decoster, J., & Cavanaugh, R. (2011). Aging prisoners’ treatment selection: Does prospect theory enhance understanding of end-of-life medical decisions? *The Gerontologist,**51*(5), 663–674. 10.1093/geront/gnr039 10.1093/geront/gnr039 PMC3218636 21593007

[CR75] Powell, T. E., Boomgaarden, H. G., De Swert, K., & de Vreese, C. H. (2015). A clearer picture: The contribution of visuals and text to framing effects. *Journal of Communication,**65*(6), 997–1017. 10.1111/jcom.12184

[CR76] Rasiel, E. B., Weinfurt, K. P., & Schulman, K. A. (2005). Can prospect theory explain risk-seeking behavior by terminally Ill patients? *Medical Decision Making,**25*(6), 609–613. 10.1177/0272989X05282642 16282211 10.1177/0272989X05282642

[CR77] Ratcliff, R., & Childers, R. (2015). Individual differences and fitting methods for the two-choice diffusion model of decision making. *Decision,**2*, 237–279. 10.1037/dec0000030 10.1037/dec0000030 PMC4517692 26236754

[CR78] Ratcliff, R., Thompson, C. A., & McKoon, G. (2015). Modeling individual differences in response time and accuracy in numeracy. *Cognition,**137*, 115–136. 10.1016/j.cognition.2014.12.004 25637690 10.1016/j.cognition.2014.12.004 PMC4353499

[CR79] Reiner, P. B. (2019). Experimental neuroethics. *Advances in Neuroethics* (pp. 75–83). Springer International Publishing.

[CR80] Riis, J., Simmons, J. P., & Goodwin, G. P. (2008). Preferences for enhancement pharmaceuticals: The reluctance to enhance fundamental traits. *Journal of Consumer Research,**35*(3), 495–508. 10.1086/588746

[CR81] Rossi, S., Antal, A., Bestmann, S., Bikson, M., Brewer, C., Brockmöller, J., . . . et al. (2021). Safety and recommendations for TMS use in healthy subjects and patient populations, with updates on training, ethical and regulatory issues: Expert guidelines. *Clinical Neurophysiology,**132*(1), 269–306. 10.1016/j.clinph.2020.10.003 PMC9094636 33243615

[CR82] Sample, M., Sattler, S., Blain-Moraes, S., Rodríguez-Arias, D., & Racine, E. (2019). Do publics share experts’ concerns about brain–computer interfaces? A trinational survey on the ethics of neural technology. *Science, Technology & Human Values*. 10.1177/0162243919879220

[CR83] Schelle, K. J., Faulmüller, N., Caviola, L., & Hewstone, M. (2014). Attitudes toward pharmacological cognitive enhancement—a review. *Frontiers in Systems Neuroscience,**8*. 10.3389/fnsys.2014.00053 10.3389/fnsys.2014.00053 PMC4029025 24860438

[CR84] Scheske, C., & Schnall, S. (2012). The ethics of “smart drugs’’: Moral judgments about healthy people’s use of cognitive-enhancing drugs. *Basic and Applied Social Psychology,**34*(6), 508–515. 10.1080/01973533.2012.711692

[CR85] Schubert, A.-L., Nunez, M. D., Hagemann, D., & Vandekerckhove, J. (2019). Individual differences in cortical processing speed predict cognitive abilities: A model-based cognitive neuroscience account. *Computational Brain & Behavior,**2*(2), 64–84.

[CR86] Schwartz, A., Goldberg, J., & Hazen, G. (2008). Prospect theory, reference points, and health decisions. *Judgment and Decision Making,**3*(2), 7.

[CR87] Soekhai, V., de Bekker-Grob, E. W., Ellis, A. R., & Vass, C. M. (2019). Discrete choice experiments in health economics: Past present and future. *PharmacoEconomics,**37*(2), 201–226. 10.1007/s40273-018-0734-2 30392040 10.1007/s40273-018-0734-2 PMC6386055

[CR88] Soto, C. J., & John, O. P. (2017). The next big five inventory (BFI-2): Developing and assessing a hierarchical model with 15 facets to enhance bandwidth, fidelity, and predictive power. *Journal of Personality and Social Psychology,**113*(1), 117–143. 10.1037/pspp0000096 10.1037/pspp0000096 27055049

[CR89] Specker, J., Schermer, M. H. N., & Reiner, P. B. (2017). Public attitudes towards moral enhancement. Evidence that means matter morally. *Neuroethics,**10*(3), 405–417. 10.1007/s12152-017-9340-9 28890740 10.1007/s12152-017-9340-9 PMC5569135

[CR90] Thaler, R. H., Tversky, A., Kahneman, D., & Schwartz, A. (1997). the effect of myopia and loss aversion on risk taking: An experimental test*. *The Quarterly Journal of Economics,**112*(2), 647–661. 10.1162/003355397555226

[CR91] Thomas, K. A., & Clifford, S. (2017). Validity and Mechanical Turk: An assessment of exclusion methods and interactive experiments. *Computers in Human Behavior,**77*, 184–197. 10.1016/j.chb.2017.08.038

[CR92] Treadwell, J. R., & Lenert, L. A. (1999). Health values and prospect theory. *Medical Decision Making,**19*(3), 344–352. 10.1177/0272989X9901900313 10424841 10.1177/0272989X9901900313

[CR93] Ubel, P. A., Angott, A. M., & Zikmund-Fisher, B. J. (2011). Physicians recommend different treatments for patients than they would choose for themselves. *Archives of Internal Medicine,**171*(7), 630–634. 10.1001/archinternmed.2011.91 21482835 10.1001/archinternmed.2011.91 PMC3817828

[CR94] van Osch, S. M. C., van den Hout, W. B., & Stiggelbout, A. M. (2006). Exploring the reference point in prospect theory: Gambles for length of life. *Medical Decision Making,**26*(4), 338–346. 10.1177/0272989X06290484 16855123 10.1177/0272989X06290484

[CR95] Velligan, D. I., Sajatovic, M., Hatch, A., Kramata, P., & Docherty, J. P. (2017). Why do psychiatric patients stop antipsychotic medication? A systematic review of reasons for nonadherence to medication in patients with serious mental illness. *Patient preference and adherence,**11*, 449–468. 10.2147/PPA.S124658 28424542 10.2147/PPA.S124658 PMC5344423

[CR96] Walsh, V., & Pascual-Leone, A. (2003). *Transcranial magnetic stimulation: a neurochronometrics of mind*. MIT press.

[CR97] Waters, E. A., Weinstein, N. D., Colditz, G. A., & Emmons, K. M. (2007). Reducing aversion to side effects in preventive medical treatment decisions. *Journal of Experimental Psychology: Applied,**13*(1), 11–21. 10.1037/1076-898X.13.1.11 17385998 10.1037/1076-898X.13.1.11

[CR98] Wickham, H. (2009). Elegant graphics for data analysis. *Media,**35*(211), 10–1007.

[CR99] Winter, L., & Parker, B. (2007). Current health and preferences for life-prolonging treatments: An application of prospect theory to end-of-life decision making. *Social Science & Medicine,**65*(8), 1695–1707. 10.1016/j.socscimed.2007.06.012 10.1016/j.socscimed.2007.06.012 17655996

[CR100] Zhang, Y., Méndez, S. J., & Scott, A. (2019). Factors affecting general practitioners’ decisions to adopt new prescription drugs – Cohort analyses using Australian longitudinal physician survey data. *BMC Health Services Research,**19*(1), 94. 10.1186/s12913-019-3889-4 10.1186/s12913-019-3889-4 PMC6366109 30728010

